# CLE peptides act via the receptor-like kinase CRINKLY 4 in *Physcomitrium patens* gametophore development

**DOI:** 10.1080/15592324.2024.2386502

**Published:** 2024-07-31

**Authors:** Alain Shumbusho, C. Jill Harrison, Viktor Demko

**Affiliations:** a Faculty of Natural Sciences, Department of Plant Physiology, Comenius University in Bratislava, Bratislava, Slovak Republic; b School of Biological Sciences, University of Bristol, Bristol, UK; c Plant Science and Biodiversity Center, Slovak Academy of Science, Bratislava, Slovak Republic

**Keywords:** Cell-to-cell signaling, CLAVATA, leaf development, *Physcomitrium patens*, crinkly4 kinase, DEFECTIVE KERNEL

## Abstract

The CLAVATA pathway plays a key role in the regulation of multicellular shoot and root meristems in flowering plants. In Arabidopsis, CLAVATA 3-like signaling peptides (CLEs) act via receptor-like kinases CLAVATA 1 and CRINKLY 4 (CR4). In the moss *Physcomitrium patens*, PpCLAVATA and PpCR4 were previously studied independently and shown to play conserved roles in the regulation of cell proliferation and differentiation. The plant calpain DEFECTIVE KERNEL 1 (DEK1) has been identified as another key regulator of cell division and cell fate in vascular plants and bryophytes. The functional interaction between CLAVATA, CR4, and DEK1 remains unknown. Here, we show that *P. patens crinkly4* and *dek1* mutants respond differently to CLE peptide treatments suggesting their distinct roles in the CLAVATA pathway. Reduced CLAVATA-mediated suppression of leafy shoot growth in *Δcr4* mutants indicates that PpCR4 is involved in CLV3p perception, most likely as a receptor. The CLV3p strongly suppressed leaf vein development in *Δcr4* mutants, suggesting that other receptors are involved in these processes and indicating a potential role of PpCR4 in organ sensitization to CLEs.

## Introduction

Cell-to-cell communication plays a critical role in the differentiation and maintenance of distinct cell layers, consequently affecting multicellular body size and shape. In plants, the CLAVATA pathway represents a receptor kinase:ligand-based intercellular signaling mechanism that governs multiple developmental programs, including the organization of multicellular shoot and root apical meristems.^1–6^ Arabidopsis CLAVATA signaling involves CLAVATA 1 (CLV1) and CLV2 leucine-rich repeats kinases that act as receptors to the CLV3 signal peptide. The family of CLV3-like peptides comprises 32 members in *Arabidopsis thaliana*,^7,8^ acting through diverse receptor kinases.^2,9,10^ For instance, CLV3 peptides signal via RECEPTOR-LIKE PROTEIN KINASE 2/TOADSTOOL 2 (RPK2/TOAD2) homodimers and heteromultimers of CLV1 with its homologs BARELY ANY MERISTEM 1 (BAM1) and BAM2.^11–13^ CLAVATA signaling controls spatial restriction of the WUSCHEL transcription factor (WUS), one of the key regulators of stem cell activity in plant shoot apices.^14,15^ Beyond the shoot apical meristem, roles of CLV1, CLV2, and CLEs in leaf and fruit development have been reported.^16^ In roots, CLEs act via CLV1, CLV2, and the tumor necrosis factor-like receptor kinase CRINKLY 4 (CR4) to confine WUS-related homeobox transcription factor expression and thus spatially control root apical meristem organization.^2,4,17^ Originally, CR4 function was identified in maize, where it affects the epidermis and endosperm aleurone layer differentiation.^18,19^ Following studies in monocots and dicots shown that CR4 kinase indeed plays an important role in epidermal (L1) layer formation in diverse organs.^20–23^ In *A. thaliana*, heterocomplexes of CLV1 and CR4 (also known as Arabidopsis CRINKLY 4, ACR4) have been described to control stem cell specification in root apical meristem.^17^ ACR4, and its homologs in monocots, have been implicated in surrounding tissue formation such as ovule integuments, endosperm aleurone layer, and leaf epidermis.^20,22,24–27^ The membrane-anchored calpain protease DEFECTIVE KERNEL 1 (DEK1) has been identified as a key regulator of division plane orientation, stem cell formation, and epidermal cell fate specification.^23,26,28–30^ DEK1 and CR4 colocalized to plasma membrane and endosomes together with a class E vacuolar sorting protein SAL1.^26^

Genetic analyses in the moss *Physcomitrium patens*, a bryophyte sister of vascular plants, shed more light on the evolutionary developmental aspects of CLAVATA signaling. Unlike flowering plant sporophytes that have multilayered meristems, single apical stem cells contribute to body formation in moss gametophytes.^31^ In *P. patens*, the life cycle begins with a haploid spore that germinates to produce filamentous protonemata. Protonemata form branched filaments composed of caulonema and chloronema cells.^32,33^ Distinct protonemal side-branched initial cells acquire different identities^34^ and after a series of precisely oriented asymmetric divisions form a bud with an apical stem cell giving rise to a leafy gametophore.^35^ *P. patens* phyllids (for simplicity hereafter called leaves) are composed of a single cell-layered lamina, and a more complex midrib composed of specialized supporting and water-conducting cells.^36,37^ Reproductive organs (gametangia) are formed at the gametophore apex, where after fertilization, a sporophyte develops, producing haploid spores to close the life cycle.^38,39^

The *P. patens* CLAVATA pathway involves CLV3-like peptides encoded by *PpCLE1–9* genes and PpCLV1a, PpCLV1b, and PpRPK2 receptor-like kinases.^40^ Based on genetic analyses, it was proposed that PpRPK2 regulates the distribution of the plant hormone auxin, thereby affecting stem cell activity and growth in filamentous protonemata.^41^ CLAVATA plays a critical role during the transition from filamentous to complex three-dimensional growth by controlling oriented cell divisions leading to gametophore stem cell formation.^40^ Downregulation of genes encoding PpCLE peptides as well as loss-of-function of PpCLV1 and PpRPK2 led to uncontrolled cell proliferation and developmental defects during vegetative^40,42^ and reproductive^43^ development. The role of CLAVATA in the regulation of cell division plane orientation and cell proliferation is conserved between *P. patens* and *Arabidopsis thaliana* as revealed by comparative analyses of *clv/bam* mutants and cross-sensitivity to applied CLE peptides from both species.^40^ A recent gene regulatory network analysis in *P. patens* identified a PpDEK1-controlled regulon affecting the CLAVATA pathway.^44^ In *P. patens*, deletion of PpCR4 caused developmental defects in leafy gametophores, reproductive organs, and sporophytes.^45^ Despite its critical role as a vital component of the CLAVATA pathway within root apices of Arabidopsis, the functional association between CR4 and CLEs in upper ground organs of angiosperms and in bryophytes remains unclear.

Here, we investigated whether PpCR4 and PpDEK1 act with CLAVATA to regulate leafy gametophore development in *P. patens*. We tested the effect of applied CLV3 and PpCLE peptides on *P. patens* plants lacking PpCR4 (*Δcr4*) and a mutant with modified PpDEK1 function (*dek1*_*Δlg3*), each with distinctly affected leaf development. The results indicate that CLE peptides specifically affect gametophore development in the absence of PpCR4 kinase, causing developmental arrest of leaves at their juvenile (basal) state.

## Material and methods

The Gransden wild-type (WT) strain of *Physcomitrium patens*,^46^ the *Δcr4* mutant strain,^45^ and *dek1_ Δlg3* mutant strain^47^ were used (all strains available in V.D. lab from previous works). Plant material was cultivated on sterile BCDAT media at 23°C in continuous light at 30–50 µmol m^−2^ s^−1^ photosynthetically active radiation in Sanyo MLR-351 growth cabinets. Synthetic CLE peptides (Genecust, >95% purity, CLV3: RTVPSGPDPLHH, PpCLE1,2,3: RMVPTGPNPLHN, and Random peptides: PHHLPGPSTRDV)^40^ were dissolved in phosphate buffer (50 μM, pH 6, 8) to stock concentration of 10 mM and supplemented to BCDAT media to final concentration of 10 μM. To assess whole plant and gametophore phenotypes, 4-week-old spot cultures were imaged using a Keyence VHX-1000E digital microscope with a 20–50× or 50–200× objective. Procreate and ImageJ 1.54f softwares allowed cell counting, area quantification, and heatmapping. Biorender.com Beta version was used for data representation. Analysis of variance was performed using the STATGRAPHICS CENTURION V. 15 software. Boxplots show interquartile range with the median at the center and delimited by minimum and maximum values that are not outliers. The bar graphs represent the mean and the standard error of the mean (SE).

## Results and discussion

### *crinkly4* and *dek1* mutants are sensitive to CLV3-like peptides

**Figure 1.**
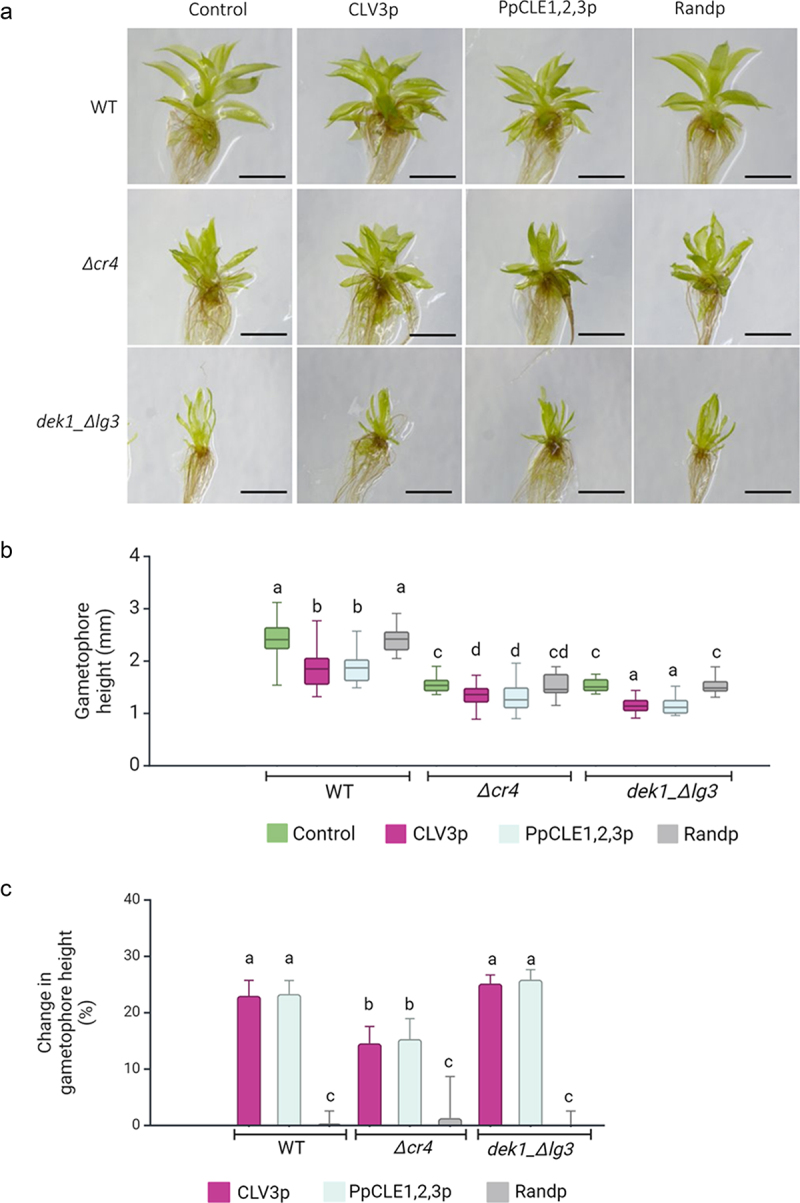
The reduced gametophore height response to CLV3 and PpCLE1,2,3 peptides depends on PpCR4.

*P. patens* WT plants, mutants with a deleted *PpCR4* gene (*Δcr4*), and mutants lacking the LG3-like domain in PpDEK1 (*dek1_ Δlg3*) were treated with CLV3 peptide (CLV3p), PpCLE1,2,3p, and random peptides, respectively (Figure 1a). In WT plants, the growth of gametophores was reduced in response to CLV3p (23.01%) and PpCLE1,2,3p (23.32%) (Figure 1b,c), while no significant change in the overall morphology and gametophore height was determined in the presence of control random peptides. *Δcr4* mutant gametophores are smaller than WT gametophores but showed a further growth reduction upon CLV3p treatment (14.57%) as well as PpCLE1,2,3 treatment (15.34%). No significant change in gametophore height was determined in the presence of random peptides (Figure 1a-c). Gametophore development in *dek1_ Δlg3* mutant is severely affected compared to WT (for details, see Johansen et al., 2016),^47^ with reduced stem height and narrow leaves. Nevertheless, CLE3p and PpCLE1,2,3p caused further 25.19% and 25.88% gametophore height reduction respectively, and these mutants thus showed a normal response to CLEs (Figure 1a-c). The relative reduction of CLAVATA-mediated suppression of gametophore height in *Δcr4* mutants (Figure 1c) indicates that PpCR4 is required in peptide signaling, most likely acting as a receptor to CLEs.

(a) Isolated gametophores from 1-month-old cultures grown on control media, and media supplemented with CLV3p, PpCLE1,2,3p, and Random peptides, respectively. Scale bars: 1 mm. (b) Height measured from ≥20 gametophores. (c) Percentage change in gametophore height response to CLV3p, PpCLE1,23p, and Randp. The analysis of variance was performed, and means were separated using the least square difference (LSD) post hoc test. Statistical significance: <5%. Values with similar letters are statistically not significant. Data in (c) represent the mean ± SE.

### CLV3 peptides differently affect leaf morphogenesis in Δcr4 and dek1_Δlg3 mutants

The PpDEK1 calpain protease is essential for early asymmetric cell divisions that give rise to the gametophore apical stem cell.^30,48^ Based on recent gene regulatory network analyses, it has been proposed that DEK1 mediates positional signaling that involves CLAVATA regulon to control phase transition from filamentous growth to three-dimensional growth. Here, PpDEK1 seems to act in a feedback control of *PpCLV1b* expression separately from a PpRPK2-mediated pathway.^44^ Previously, it has been shown that calpain activity in *dek1_ Δlg3* is sufficient to maintain gametophore apical stem cell; however, it fails to maintain cell proliferation during leaf development.^47^ Morphologically, the leaves of *dek1_ Δlg3* mutant were not significantly changed after CLV3p treatment, which supports upstream-acting and fine-tuning role of PpDEK1 during leaf morphogenesis. However, CLV3 and PpCLE1,2,3 peptide treatments caused specific morphological changes in *Δcr4* leaves.

In WT *P. patens* plants, the leaves are morphologically different depending on their position along the apical-basal axis of gametophore, a phenomenon known as heteroblasty.^49,50^ The earliest developed juvenile leaves at the gametophore base are morphologically simpler compared to successively formed leaves positioned toward the apex. They are oblong in shape and mostly formed of a single-layered lamina, lacking a vein. The transition to leaves positioned toward the middle of gametophores is associated with the formation of a vein and development of elongated marginal serrated cells. Cell size along the longitudinal axis of mature leaves also varies with elongated cells at the base and isodiametric cells toward the apex. Newly formed apical leaves contain small proliferating cells at the base and elongated cells toward the tip. Closer examination of isolated leaves from *Δcr4* and *dek1*_*Δlg3* mutants revealed distinct responses to exogenous peptides when compared to WT plants. As the effects of both CLV3p and PpCLE1,2,3p were similar, we focused on CLV3p-treated plants for more detailed observations.

**Figure 2.**
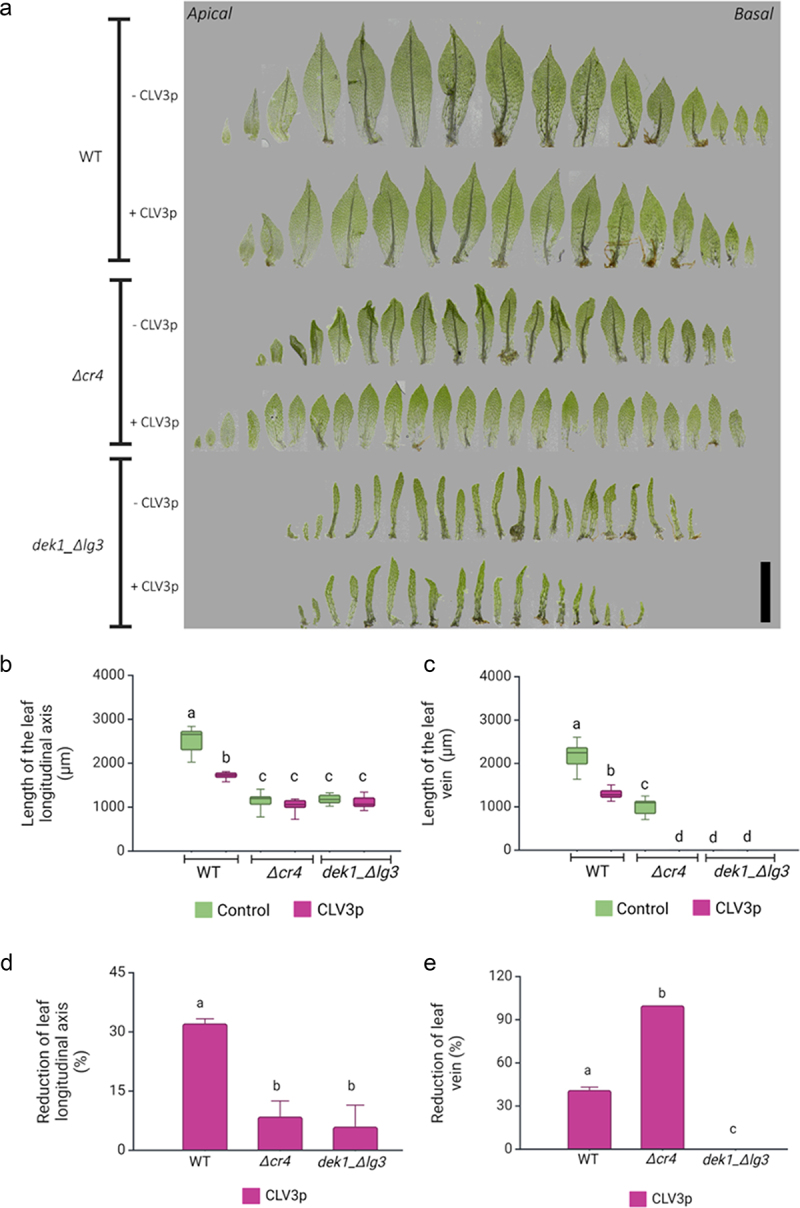
CLV3p treatment suppresses vein development *Δcr4* mutants.

While the CLV3p treatment reduced the leaf size by 30% in WT plants and there was a 41% reduction of vein length in treated leaves, overall heteroblasty was not affected (Figure 2). However, leaf development was strongly affected by CLV3p treatment in *Δcr4* mutants when compared to untreated plants. The leaves of *Δcr4* mutants lack elongated marginal cells, which likely disturb mechanical cues along the leaf axis leading to characteristic crinkliness, but veins form similarly in mutants to WT leaves (for details, see Demko et al., 2016).^45^ CLV3p treatment caused a complete loss of crinkliness in *Δcr4* leaves (Figure 2a). There was a c. 7% decrease in leaf size following treatment (Figure 2b,d) and all treated *Δcr4* leaves lacked defined midribs, resembling basal juvenile leaves (Figure 2a,c,e). This suggests that PpCR4 is required for a normal response to CLE3p treatment in leaves, and that CLEs signal through another receptor to suppress vein development.

(a) Isolated leaves from WT, *Δcr4*, and *dek1_ Δlg3* strains treated with CLV3p. Scale bar: 1 mm. (b) Longitudinal length measured from ≥10 leaves per strain. (c) Length of the leaf vein measured from ≥10 leaves per strain. (d) Percentage differences in leaf lengths after the CLV3p treatment. (e) Percentage differences in vein length after the CLV3p treatment. The analysis of variance was performed, and means were separated using the least square difference (LSD) post hoc test. Statistical significance: <5%. Values with similar letters are statistically not significant. Data in (d) and (e) represent the mean ± SE.

### Cell proliferation is suppressed by CLV3p in Δcr4 but not in dek1_ Δlg3 mutant’s leaves

**Figure 3.**
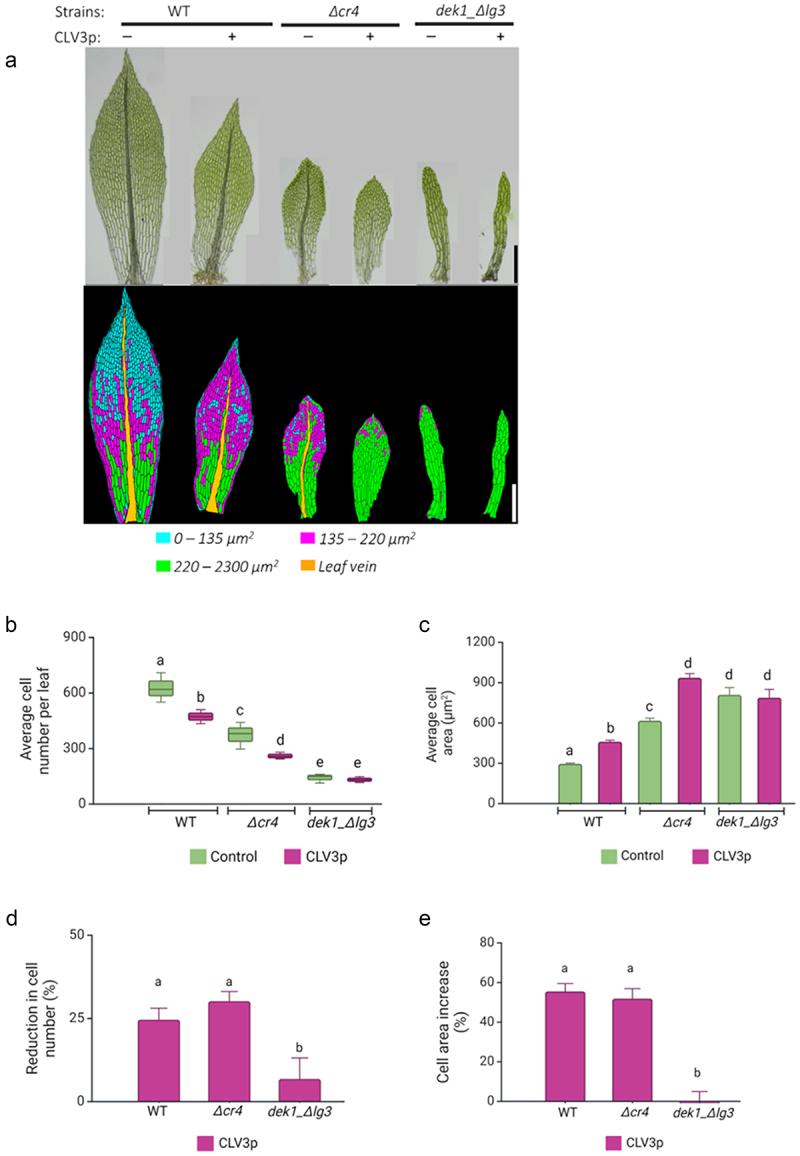
CLV3 peptides suppress cell proliferation in WT and *Δcr4* mutant plants.

Normal heteroblastic leaf development requires spatially controlled cell proliferation and elongation along the axes of leaf symmetry.^49,51^ Therefore, we next determined the effect of CLV3p treatment on leaf cell number and cell area (Figure 3, S1). In WT plants, the overall cell number was reduced upon the treatment as in previous work^40^ (Figure 3a,b). The number of cell files along the medial-lateral axis decreased, and there were fewer cells along the apical-basal axis, especially within the distal parts of the leaves (Figure S2). In contrast, the cell area increased toward the tip of the leaves (Figure 3a,c; S1). Leaf development is already strongly affected in *dek1_ Δlg3* mutant (Figure 2a) as previously described by Johansen et al. 2016,^47^ and CLV3p treatment had no significant effect on leaf size in *dek1_ Δlg3* mutant when compared to untreated plants (Figure 2). In the *Δcr4* mutant, overall leaf cell numbers decreased following CLV3p treatment (Figure 3a,b). The number of cell files along the medial-lateral and apical-basal axis decreased significantly (Figure S2). In addition, CLV3p-treated *Δcr4* leaves lost a typical distribution of cells with different cell size ranges along the apical-basal axis (Figure 3a), and cell area significantly increased throughout the midrib-less leaves (Figure 3a,c; Figure S1). Feeding WT and *Δcr4* with CLV3p resulted in a similar rate of overall cell number reduction (approx. 25%; Figure 3d) and cell area increase (approx. 50%; Figure 3e). While gametophore length decreased upon CLV3p treatment in *dek1_ Δlg3* mutant, the overall leaf size was not significantly changed (Figure 2b). The average cell number and cell area in mature *dek1_ Δlg3* leaves were not significantly affected by added CLV3p (Figure 3; Figure S1, S2). This suggests that CLAVATA pathway requires sufficient DEK1 activity to control cell proliferation during leaf development.

(a) Upper part: representative images of mature leaves dissected from WT, *Δcr4*, and *dek1*_*Δlg3* gametophores treated (+) and not treated (-) with CLV3p. Lower part: distribution of cells with color-coded areas. Scale bars: 300 μm. (b) Average cell numbers measured from three mature leaves (shown in Figure S1). (c) Average cell areas measured from leaves as in (b). (d) Percentage differences in the number of cells after the CLV3p treatment. (e) Percentage differences in cell area after the CLV3p treatment. The analysis of variance was performed, and means were separated using the least square difference (LSD) post hoc test. Statistical significance: <5%. Values with similar letters are statistically not significant. Data in (c), (d), and (e) represent the mean ± SE.

As previously demonstrated, *P. patens* CLV3-like peptides (PpCLEs) can act via conserved receptor kinases in *A. thaliana*.^40^ Protein–protein interactions between PpCR4 with PpCLV1 and PpRPK2 remain to be investigated. As we show here, the CLV3p-mediated repression of cell proliferation is exacerbated in the *Δcr4* mutant leading to loss of vein development. Hypothetically, the lack of PpCR4 might affect the assembly of interacting receptor kinase complexes and thereby their sensitivity to CLEs. In *A. thaliana* roots, the ACR4 forms heterodimers with CLV1^17^ and contributes to spatial restriction of asymmetric cell divisions in columella stem cells as well as in pericycle during lateral root initiation.^27^ In addition, the ACR4 expression domain is expanded by CLE40 peptide treatment.^16^ In *A. thaliana* embryos, both TOAD2 (RPK2) and ACR4 localize in protodermal cells, and both play important role in epidermal identity maintenance.^52,53^ In addition, TOAD2 is required for PIN1 expression and auxin distribution during early embryogenesis in *A. thaliana*.^52^ The physical interaction between PpCR4 and PpCLV1 has not been demonstrated yet. It would be also interesting to test whether PpCR4 expression is affected by CLEs in different tissues. Altogether, based on the data presented here, we propose that PpCR4 is likely a receptor for CLV3-like peptides in *P. patens* and together with other receptor kinases contributes to CLAVATA signaling during gametophyte development.^45^

## Supplementary Material

Corrected_Sup_Figure_2.tif

Corrected_Sup_Figure_1_TIFF.tif

## Data Availability

All data generated during this study are included in this article and associated supplementary files. The mutant lines presented in this study are available upon request.
