# Supplementary figures and images for: CLE peptides act via the receptor-like kinase CRINKLY 4 in Physcomitrium patens gametophore development
Source: Plant Signal Behav. 2024 Jul 31;19(1):2386502. doi: 10.1080/15592324.2024.2386502 (PMC11296525; doi:10.1080/15592324.2024.2386502)

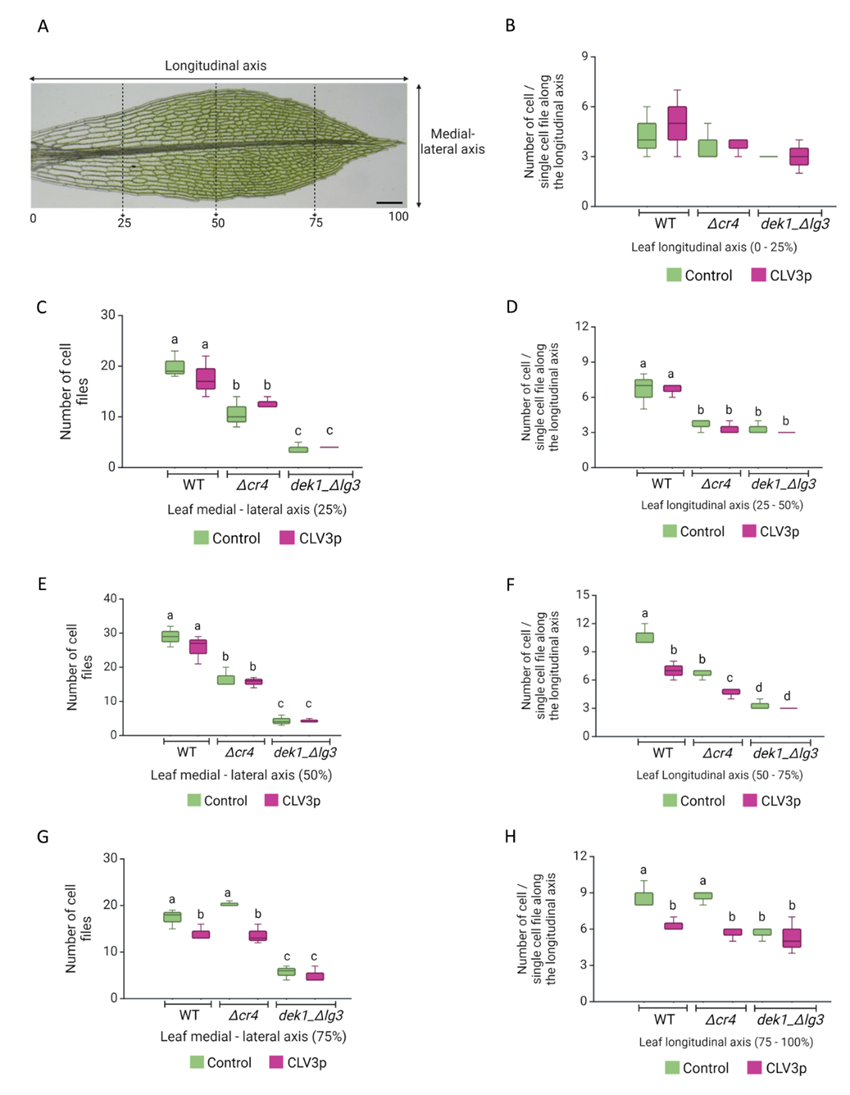

Supplement: Corrected_Sup_Figure_2.tif [file KPSB_A_2386502_SM4282.tif]

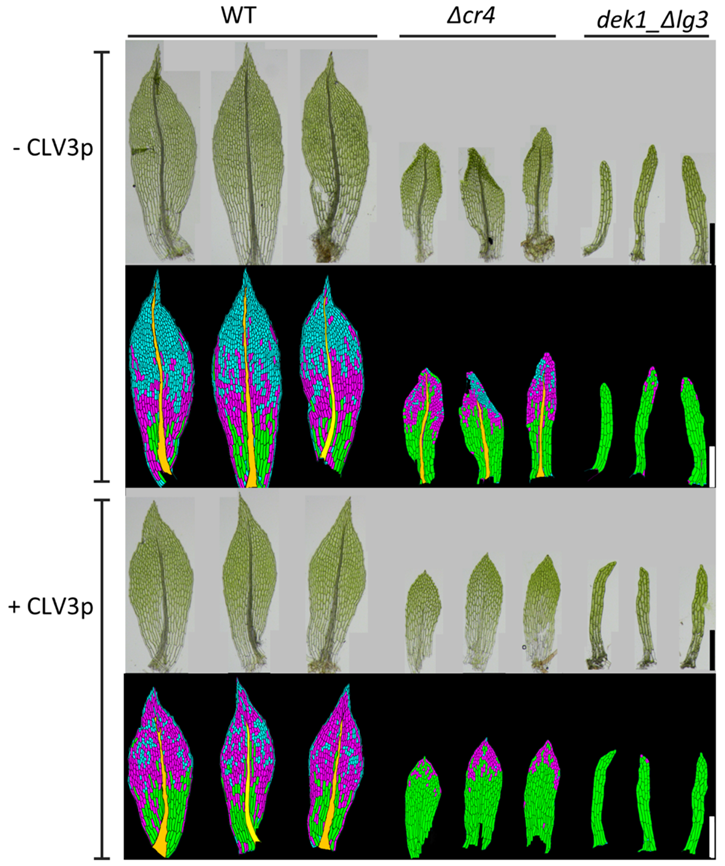

Supplement: Corrected_Sup_Figure_1_TIFF.tif [file KPSB_A_2386502_SM4281.tif]
